# Comparative analysis of outcomes between anemic and non-anemic critically ill elderly patients in a geriatric ICU in Egypt: A focused study

**DOI:** 10.2478/jccm-2025-0028

**Published:** 2025-07-31

**Authors:** Manar Mamdouh Abd Al Kader, Manar Mostafa Adel Maamoun, Walaa W. Aly, Heba Youssif Youssif, Mennatallah Safwat Elaraby

**Affiliations:** Geriatrics and Gerontology Department, Faculty of Medicine, Ain Shams University

**Keywords:** anemia, critically ill, elderly, hospital acquired infections, in-hospital mortality

## Abstract

**Background:**

Numbers of elderly patients who are being admitted to the intensive care unit (ICU) are increasing; Among ICU patients, elderly patients represent a particular subgroup, with a proportion of up to 50% for patients aged 65 years and over, and on average about 35% of admissions for patients older than 70–75 years. Also, those aged 80 years and older represent around 15% of total ICU population. In Egypt, a study conducted in seven regions found that geriatric patients represent around 48.5% of total ICU admission. Elderly individuals are more susceptible to anemia due to multiple comorbidities and age related changes. Anemia is a common problem among critically ill elderly patients with serious consequences. It is recognized as an independent risk factor for increased mortality and morbidity. In fact, anemia is the most prevalent hematologic disorder in the ICU. The prevalence of anemia among critically ill patients admitted to the ICU ranges from 60 to 66%. Approximately 60% of critically ill patients are anemic at admission, and an additional 40–50% develop anemia during their ICU stay. The condition is particularly common among older patients. Low hemoglobin (Hb) concentrations are associated with prolonged ICU and hospital stays, as well as increased mortality rates. Therefore, anemia is consequently a significant public health issue from the medical and economic perspectives.

**Aim:**

To compare outcomes between anemic and non-anemic critically ill elderly patients admitted to the Geriatric ICU at Ahmed Shawky geriatric Hospital, Ain Shams University hospitals.

**Subjects and methods:**

A Prospective cohort study was conducted on two hundred sixteen elderly patients of both sexes aged 60 years old or older. It was carried out in the geriatric ICU at Ahmed Shawky geriatric Hospital, Ain Shams University Hospitals. Data collection included participants demographics, medical history, full labs assessment and anemia evaluation based on hemoglobin level, Severity of illness was assessed by validated scoring systems, including the Sequential organ failure assessment (SOFA score) on the first day of admission, as well as Acute physiology and chronic Health Evaluation (APACHE II, APACHE IV). Additionally, the Mortality Probability Model Score (MPM0-III) was applied at first day of admission, 48hours and 72 hours following ICU admission. Anemia management strategies were documented, including the use of blood transfusions, iron therapy and other supportive treatments. Clinical outcomes assessed included ICU length of stay, Site of discharge, in-hospital Mortality and the incidence of Hospital acquired infections.

**Results:**

On admission 172(79.6%) of studied subjects were anemic, (90)41.7% had mild anemia, 56(25.9%) had moderate anemia and 26(12%) had severe anemia. Anemic patients showed significantly higher SOFA, MPM 24hrs, MPM 48hrs, MPM 72hrs, APACHE4, SAPSIII, extended hospital stays, and increased rates of hospital acquired infections(P<0.05). Predicators of mortality included the severity of anemia, the need for mechanical ventilation, and thrombocytopenia (P<0.001). However, anemia on admission to ICU was not a direct predictor of in-hospital mortality. Regarding management of anemia: seventy three (33.9%) of the anemic subjects received blood transfusion. Fourteen (6.5%) received either enteral or parental iron therapy, 20(9.3%) of studied subjects were given erythropoietin, 3(1.4%) of them were given vitamin B12 and folic acid.

**Conclusion:**

On admission, 79.6% of critically ill elderly patients had anemia. The presence of anemia in this population was associated with prolonged ICU stays, increased in-hospital mortality and a higher risk of hospital acquired infections.

## Introduction

At any age, anemia is a common disease, but this is particularly true problem among the older population since the prevalence of anemia increases with age [1]. The world Health Organization defines anemia as a hemoglobin level below 13 grams per deciliter (g/dl) in males and below 12 g/dl in females [2]. Anemia exists in 60–66% of critically ill patients admitted to ICU [3, 4] and 40–50% experience anemia during their ICU stay [5]. The number of older patients being admitted to the ICU is rising; among ICU patients, elderly patients represent a particular subgroup [6]. Elderly individuals can also be at increased risk of anemia due to comorbidities, including gastric atrophy triggering malabsorption of nutrients, gastrointestinal blood loss, myelodysplastic syndromes, and chronic conditions (e.g. chronic inflammation or chronic kidney disease) [7]. Anemia is associated with higher mortality rates and longer stays in the ICU [8]. Anemia in critically ill patients, contributes to an increase in mortality rate [3,9, 10,11] myocardial infraction, mechanical ventilation duration and length of stay [9]. In critically ill patients, anemia within the first week was associated with high risk of long-term mortality [12]. Among older persons, anemia is associated with a higher risk of death, disability [13, 14] and hospitalizations [12]. Also, it was found that anemic patients had a longer hospital stay than non-anemic patients, regardless of the cause of hospital admission, and anemic patients were more likely to be readmitted to the hospital than non-anemic patients [15]. Anemia has been identified as a risk factor for nosocomial infections among patients [16]. Also, it was an independent risk factor for in-hospital mortality from nosocomial infections [17]. It was found that anemia increased the risk of hospital acquired pneumonia [18]. The existence of anemia may impair weaning from ventilatory support and raise the risk of ventilator associated pneumonia (VAP) among patients requiring long-term mechanical ventilation [19]. Anemia was significantly associated to development of Pressure ulcers in ICU [20]. These ulcers are linked to increase risk of infection, disability, high level of dependence, longer hospital stay and higher hospitalization costs [21]. Anemia in geriatric ICU patients with nosocomial infections increases the risk of mortality [22].

The aim of the work was to compare outcomes between anemic and non-anemic critically ill elderly patients admitted to the Geriatric ICU at Ahmed Shawky geriatric Hospital, Ain Shams University hospitals.

## Methods

**Sample size:** Two hundred sixteen elderly patients of both sexes aged 60 years old and older who were admitted to the Geriatric critical care unit from (January 2023 till December 2023) then divided into anemic group and non-anemic group by hemoglobin level on admission.

**Study type:** A prospective cohort study was conducted to identify anemia in critically ill patients.

**Exclusion criteria**:

- Patients who died within the first 24 hours of admission.
- Patients readmitted twice or more within the period of study to the geriatric ICU.
- Patients lacking documented hemoglobin measurements at time of ICU admission were excluded from the study

**Inclusion criteria:**

- Patients aged 60 years or older, admitted to the Ahmed Shawky Geriatric ICU for at least 24 hours, with baseline hemoglobin measurements obtained within the first 24 hours of admission.

Were evaluated by:

- Full detailed history including medical history and assessment of comorbidities using (Charlson Comorbidity Index) [23].
- Anemia was defined and classified according to the World Health Organization (2011) criteria. In non-pregnant adults, anemia is defined as hemoglobin level (Hb): Hb < 12 g/dl in women and < 13 g/dl in men.

Different degrees of anemia were defined according to the WHO guidelines [24]:

- Mild anemia (11–11.9 g/L for women; 11–12.9 g/L for men)
- Moderate anemia (8–10.9 g/L for both sexes)
- Severe anemia (less than 8 g/L for both sexes)

**Laboratory data**:

- Hemoglobin level, white blood cell count, alanine transaminases, aspartate transaminases, total bilirubin, direct bilirubin, in direct bilirubin, blood urea nitrogen, serum creatinine were recorded.

**Assessment of severity of illness and predication of mortality by:**

- Using Sequential organ failure assessment (SOFA) score was calculated on admission and every 48 hours till discharge to predict mortality in ICU patients [25]. Mean SOFA score was calculated and maximum SOFA (SOFA max) score was recorded.
- Acute physiology and chronic health evaluation (APACHE II and APACHE IV) was calculated on admission to predict the prognosis in patients receiving intensive care using MDCalc medical calculator for APACHE II [26] and APACHE IV [27].
- Mortality Probability Model Score (MPM0-III) by using RNSH-ICU calculators on 24, 48 and 72 hours [28].
- Acute Physiology Score (SAPS 3) by using MDCalc Medical calculator on admission [29]

### Assessment of outcomes

- Length of ICU and hospital stays.
- Site of discharge: Home, Nursing home, other hospital, others
- Mortality, Time and place or DAMA. (Discharge against medical advice)
- 30 Day Mortality (In-hospital mortality).
- Hospital acquired infections: as defined by the CDC; include urinary tract infections, hospital-acquired pneumonia, ventilator-acquired pneumonia, diarrhea, and device-related infections, such as central line infections, that begin 48–72 hours after admission in patients who are either uninfected or in the incubation period at admission [30].
- Acquiring pressure ulcers.

### Anemia management

#### Regarding Blood transfusion

According to the policy of blood transfusion used at Ahmed Shawky Hospital, accredited by General Authority for healthcare Accreditation and Regulation (GAHAR), blood cells transfusion is indicated in the following situations:

- Symptomatic anemia
- Hemoglobin level < 7 g/dL
- Considerable blood loss
- Acute coronary syndrome if hemoglobin < 10 g/dL

These indications align with common clinical practices and institutional guidelines to ensure appropriate transfusion decisions.

According to the NICE guideline NG24 [31], restrictive red blood cell transfusion thresholds are recommended for patients who require transfusions but do not present with major hemorrhage, acute coronary syndrome, or chronic anemia necessitating regular transfusions. In general, a transfusion threshold of 70 g/L (7 g/dL) is advised, with a post-transfusion hemoglobin target of 70–90 g/L. For patients with acute coronary syndrome, a slightly higher threshold of 80 g/L is considered appropriate, aiming for a post-transfusion hemoglobin range of 80–100 g/L. Additionally, clinicians are encouraged to set individualized hemoglobin thresholds for patients requiring regular transfusions due to chronic anemia.

Also, According to Association for the Advancement of Blood & Biotherapies (AABB) [32]: It recommends transfusion generally recommended when hemoglobin <7 g/dL in stable adults.

Threshold of 8 g/dL for patients with cardiovascular disease emphasizes individualized decision-making

#### Regarding Iron therapy

After discussion with the intensivist, it was clarified that Iron therapy was part of the patient’s general management prior to ICU admission and was not initiated specifically to treat anemia in critical illness. Existing evidence does not support routine intravenous iron administration in critically ill patients, particularly during the acute phase, due to limited efficacy and potential safety concerns.

### Ethical Considerations

An approval for the study was granted before starting the subject’s recruitment process. The approval was obtained from the ethics committee in Ain Shams Faculty of Medicine.

### Statistical analysis

Data were tabulated and statistically analyzed using SPSS, version 16. Quantitative variables were presented as mean and standard deviation and the independent t-test will be used to compare the two groups. Qualitative data was presented as frequency and proportion and the two groups were compared using chi-square test. P-value ≤ 0.05 was considered statistically significant.

## Results

**Fig. 1.**
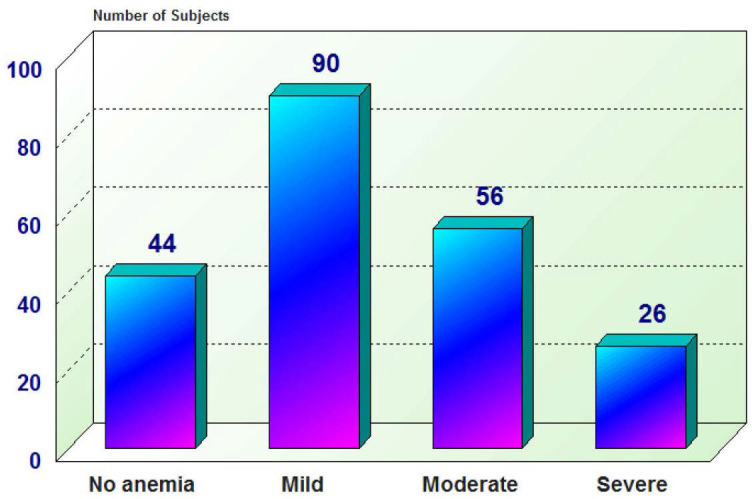
Description of grades of anemia among studied subjects

**Fig. 2.**
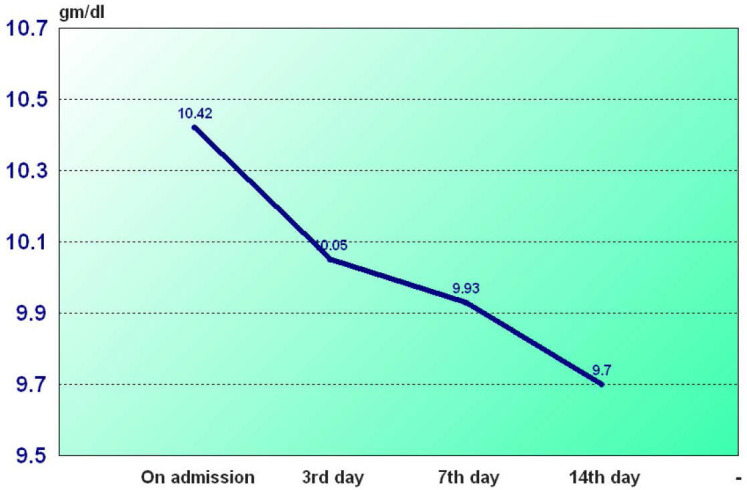
Rate of decline of hemoglobin level among studied subjects

The sample enrolled (216) consisted of 128 females and 88 males with a mean age of 75±7.84 years and the larger number of the sample were non-smokers (78.7%) as seen in (Table 1). Among the 216 studied subjects, Hypertension was the most prevalent comorbidity (122 subjects) and the least prevalent was hepatic diseases (26 subjects). Fifty subjects (23.1%) had history of previous ICU admission, subjects with Charlson Comorbidity Index which predicts 10years survival (CCI) of <7 were 140 (64.8 %) and those with index >7 were 76(35.2%) as seen in (Table 1). Among the 216 studied subjects, 172 were anemic at admission, classified into 90 had mild anemia, 56 had moderate anemia and 26 had severe anemia as seen in (Figure 1). The mean hemoglobin level on admission was 10.4 g/dl as seen in (Figure 2).

**Table 1.** Demographics, premorbid comorbidities and Charsoln comorbidity index among studied subjects (n=216)

**Quantitative:**	**Mean±SD**	**Range**
Age(yrs)	75.00±7.84	60–98

Qualitative:	No	%
Males	88	40.7
Females	128	59.3

Smoking status:		
-Non-smokers	170	78.7
-Smokers	25	11.6
-Ex-smokers	21	9.7

Premorbid Comorbidities	No	%
Hypertension	122	56.5
Cardiac diseases^1^	84	38.9
Diabetes mellitus	82	38
Dementia	73	33.8
Neurological diseases^2^	59	27.3
Renal diseases^3^	38	17.6
Pulmonary diseases^4^	31	14.4
Premorbid anemia	31	14.4
Hepatic diseases^5^	26	12

Charsoln comorbidity index:		
-less than 7	140	64.8
-More than or equal 7	76	35.2

Previous ICU admission	50	23.1

1 This includes heart failure, ischemic heart diseases, arrhythmias and valvular heart diseases

2 This includes Stroke, Seizures

3 This includes chronic kidney diseases and End Stage Renal Disease on dialysis

4 This includes Chronic obstructive pulmonary disease, Bronchial asthma, Tuberculosis

5 This includes chronic liver diseases.

Regarding outcome of the subjects in-hospital mortality (30-day mortality) 43.5% were died, ninety-six subjects were discharged to ward. Twenty-one subjects were discharged to palliative care unit and 5 subjects were discharged to home as seen in (Table 2).

**Table 2.** Description of outcomes among studied patients

**Outcomes**	**All patients (n=216)**	**%**
In hospital mortality (30 day mortality)	94	43.5

	**N = 122**	**%**
Discharge to		
-Ward	96	44.4
-Palliative Care unit	21	9.7
-Home	5	2.3

**Fig. 3.**
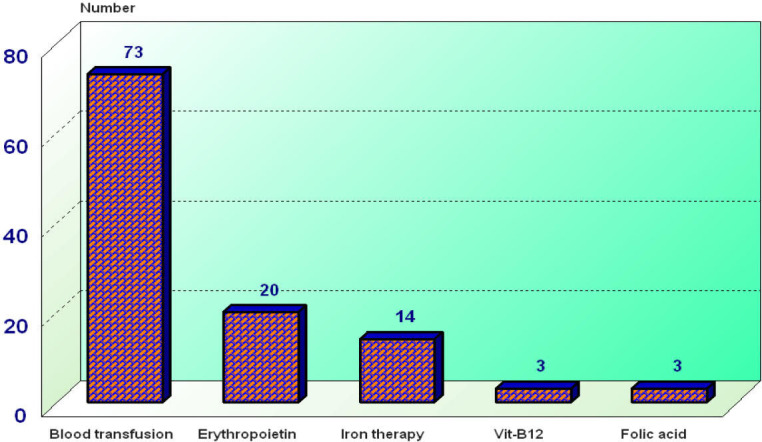
Management of anemia among studied subjects

Regarding management of anemia 73 (33.9%) of the anemic subjects received blood transfusion, 14 (6.5%) received iron therapy, 20 (9.3%) of studied subjects were given erythropoietin, 3 (1.4%) of them were given vitamin B12 and 3 (1.4 %) were given folic acid as seen in (Figure 3). As regards the comparison between anemic and non-anemic regarding age and sex, it revealed no statistically significant difference between both groups (P-value: 0.96, 0.17), anemic subjects had significantly higher SOFA and APACHE II scores (P<0.001) as seen in (Table 3).

**Table 3.** Comparison between anemic and non-anemic groups as regard age, sex, severity of illness and predictors of mortality tools

	**Anemic (n=172) (Mean+SD) (+SEM)**	**Non anemic (n=44) (Mean+SD) (+SEM)**	**Student’s “t”/Z**	**P (Sign.)**
Age (yrs)	74.99+7.78	75.05+8.15	“t”= 0.04	(0.97) >0.05(NS)

	**No (%)**	**No (%)**	**X^2^**	**P(Sign.)**
**Sex**				
Male	74 (84.1%)	14(15.9%)	1.82	(0.17) >0.05(NS)
Female	98 (76.6%)	30(23.4%)		

**Severity of illness and Predictors of mortality tools**				
Initial SOFA	20.78±18.01 (±1.37)	12.12+9.49 (+1.43)	Z=3.21	(0.001) <0.001 (HS)
Mean SOFA	52.51±32.01 (±2.44)	33.37±32.54 (±4.91)	Z=3.69	(0.001) <0.001 (HS)
Highest SOFA	25.99±30.36 (±2.31)	12.48±21.27 (±3.21)	Z=3.56	(0.001) <0.001(HS)
APACHE II	18.27±0. 51	14.05±0.92	“t”= 3.79	(0.001) <0.001(HS)
APACHE IV	71.98±19.9	63.8±15.58	“t”= 2.54	(0.012) <0.05 (S)
MPM 24 hrs	31.86±20.42 (±1.56)	22.59±15.19 (±2.29)	Z=3.08	(0.002) <0.05 (S)
MPM 48 hrs	37.79±22.73 (±1.75)	27.58± 18.92 (±2.85)	Z=2.96	(0.003) <0.05 (S)
MPM 72hrs	40.53±23.19 (±1.82)	29.45± 19.08 (±2.98)	Z=3.04	(0.002) <0.05 (S)
SAPSIII	68.11±11.61	62.78±9.16	“t”= 2.82	(0.005) <0.05 (S)

Sequential organ failure (SOFA); Acute Physiology and Chronic Health Evaluation (APACHE); Mortality Probability Model (MPM); Simplified Acute Physiology Score (SAPS)

It was found that renal disease was higher among anemic group than non-anemic group. It revealed significant increase in anemic (P<0.05). Regarding CCI was higher among anemic than non-anemic and there was statistically significant difference between them (P<0.05) as seen in (Table 4).

**Table 4.** Comparison between anemic and non-anemic as regard Comorbidity data and Charlson Comorbidity index

**Comorbidities**	**Anemic (n=172) No (%)**	**Non-anemic (n=44) No(%)**	**X^2^**	**P(Sign)**
Neurological diseases^1^	49(83.1%)	10(16.9%)	4.23	(0.44)>0.05(NS)
Hypertension	101(61.5%)	21(17.2%)	1.72	(0.18)>0.05(NS)
Pulmonary diseases^1^	28(90.3%)	3(9.7%)	2.55	(0.11)>0.05(NS)
COVID 19 infection	10(76.9%)	3(23.1%)	0.06	(0.8)>0.05(NS)
Diabetes Mellitus	67 (81.7%)	15(18.3%)	0.35	(0.55)>0.05(NS)
Cardiac disease^3^	69 (82.1%)	15 (17.9%)	0.53	(0.46)>0.05(NS)
Renal disease^4^	35 (92.1%)	3 (7.9%)	4.42	(0.03)<0.05(S)
Hepatic diseases^5^	23(88.5%)	3(11.5%)	1.42	(0.23)>0.05(NS)
Premorbid Anemia	30(96.8%)	1(3.2%)	6.55	(0.01)<0.05(S)
Dementia	56 (76.7%)	17 (23.3%)	0.57	(0.44)>0.05(NS)
Malignancy	29(90.6%)	3(9.4%)	2.80	(0.09) >0.05(NS)
Previous ICU admission	47(94%)	3(6%)	8.28	(0.004)<0.05(S)

**Charlson Comorbidity Index**				
<7	105(75%)	35(25%)	5.25	(0.02)<0.05(S)
>7	67(88.2%)	9(11.8%)		

1 This includes Stroke, Seizures;

2 This includes Chronic obstructive pulmonary disease, Bronchial asthma, Tuberculosis;

3 this includes heart failure, ischemic heart diseases, arrhythmias and valvular heart diseases;

4 This includes chronic kidney diseases and End Stage Renal Disease on dialysis;

5 This includes chronic liver diseases.

Anemic subjects had longer ICU stay than non-anemic subjects (P<0.05) as seen in (table 5), In hospital mortality was 43.5% as seen in (Table 2), with higher rates observed in the anemic group. The anemic group also showed a higher prevalence of hospital acquired pneumonia (83.9%) compared to non-anemic subjects (P<0.05) as seen in (Table 5).

**Table 5.** Comparison between anemic and non-anemic groups as regard hemodynamic support, mechanical ventilation and outcomes

	**Anemic (n=172) (Mean±SD) (±SEM)**	**Non anemic (n=44) (Mean±SD) (±SEM)**	**Student’s “t”/Z**	**P(sign.)**
Outcomes:				
Length of stay (LOS)(Days)	8.42±0.46	7.68±0.97	“t”= 0.71	0.04 <0.05(S)

	**No(%)**	**No(%)**	**X^2^**	**P(sign.)**
In hospital mortality	83(88.3)	11(11.7)	7.71	(0.05) <0.05(S)
Hospital acquired infections	118(82.5%)	25(17.5%)	2.17	(0.14) >0.05(NS)
Hospital acquired pneumonia(HAP)	78(83.9%)	15(16.1%)	10.21	(0.001) <0.05(S)

**Interventions**				
Vasopressor	36(81.8%)	8(18.2%)	0.16	(0.68) >0.05(NS)
Mechanical ventilation	28(84.8%)	5(15.2%)	0.65	(0.41) >0.05(NS)

** There was no statistically significant difference between both groups regarding Ventilator associated pneumonia (VAP), Central line associated blood stream associated infection, Diarrhea, Urinary tract infection and acquired pressure ulcers (not mentioned in table 5).

Regarding hemodynamic support and mechanical ventilation, the use of vasopressors and mechanical ventilation were higher in anemic patients. But there was no statistically significant difference between anemic and non-anemic (P>0.05) as seen in (Table 5).

A linear regression analysis examining factors influencing in-hospital mortality revealed that smoking, vasopressor use, blood transfusion, MPM at 72 hours, and elevated CRP levels were statistically significant predictors (P < 0.05). Thrombocytopenia and mechanical ventilation showed even higher statistically significance (P < 0.001) after adjusting for other variables.

In contrast, anemia at admission and its severity grades were not statistically significant factors influencing in-hospital mortality, as detailed (in Table 6).

**Table 6.** Linear regression analysis for factors affecting of mortality in ICU

	**Unstandardized B**	**Coefficients Std. Error**	**Standardized Coefficients Beta**	**t**	**P-value (Sign.)**
Age	0.006	0.004	0.088	1.51	0.13(NS)
Smoking	0.113	0.044	0.146	2.53	0.012(S)
Previous ICU admission	−0.006	0.069	−0.005	−0.08	0.93(NS)
Anemia(past history)	−0.144	0.085	−0.10	−1.68	0.09(NS)
Anemia on admission	0.137	0.092	0.111	1.48	0.14(NS)
Grades of anemia	−0.6	0.04	−0.124	−1.45	0.14(NS)
Hospital acquired anemia	0.100	0.117	0.064	0.85	0.39(NS)
Mechanical ventilation	0.33	0.092	0.24	3.62	0.00(HS)
Vasopressor	0.175	0.09	0.14	1.94	0.05(S)
Blood transfusion	0.184	0.07	0.17	2.48	0.01(S)
CRP	0.001	0.00	0.12	2.19	0.02(S)
Platelets	0.00	0.00	0.143	−2.88	0.00(HS)
MPM72 hours	0.01	0.005	0.44	2.07	0.04(S)

Dependent Variable: in hospital mortality #; Mortality Probability Model (MPM); C reactive protein (CRP); ** Other variables were statistically insignificant predicators of in hospital mortality, including: Total leucocytic count, Alkaline transaminase(ALT), Aspartate transaminase(AST), Tools of predicators of mortality including: MPM24hrs, MPM48hrs, SAPSIII, Acute Physiology and APACHEII, APACHEIV, SOFA, Initial SOFA, mean SOFA, Highest SOFA were not shown in table 6.

## Discussion

The current study was done to detect anemia in critically ill patients and to compare between anemic and non-anemic subjects in geriatric ICU regarding outcomes in the Geriatric Hospital in Ain Shams University.

The study enrolled two hundred sixteen elderly patients from both sexes mean age of all subjects was 75, One hundred twenty eight out of 216 subjects were females (59.3%).

Our findings demonstrate a high prevalence of anemia among critically ill elderly patients. In agreement with most studies, anemia is prevalent on admission among critically ill patients. In current study it was found that average hemoglobin level on admission was 10.42g/dl. Several studies showed that anemia on admission to ICU was prevalent. A met analysis conducted by Walsh and Saleh [5] reported that the mean hemoglobin at ICU admission was 10.5g/dl.

Another cohort study [3] on 3534 patients admitted to 146 ICUs in Western European established that the mean hemoglobin at ICU admission was 11.3 g /dl.

In several studies among critically ill patients the prevalence of anemia was found 46% [33], 60–66% [3,4] and 84.6% [34]. In current study it was found that 79.6% of the studied subjects were anemic on admission.

Regarding the grades of anemia at ICU admission in current study it was found that (90)41.7% had mild anemia, 56 (25.9%) had moderate anemia and 26 (12%) had severe anemia. Which is inconsistent with Walsh and Saleh [5] 25% of subjects had severe anemia (a hemoglobin level <9 g dl).

Critically ill patients admitted to the ICU represent a heterogeneous group with varying underlying conditions, making the management of anemia in this population complex and subject to ongoing debate.

Current therapeutic options include blood transfusion, iron therapy, and erythropoietin administration. In recent years, novel therapeutic modalities such as hepcidin antagonists [35], nano iron formulations [36], and IL-6 receptor antagonists [37] have emerged; however, these are not yet widely available in all settings especially in our geriatric ICU. In the current study, the most commonly used intervention for anemia management was blood transfusion, administered in 73 out of 172 patients (33.9%), followed by iron therapy in fourteen patients and erythropoietin in twenty patients. This finding is consistent with Saker et al. [38] who reported that 30.9% of 1,833 patients admitted to the ICU received a blood transfusion.

Also, Vincent et al. [39] conducted a met analysis on 730 ICUs in 84 countries and included all adult patients admitted between 8 May and 18 May 2012, excluding those admitted for postoperative monitoring. Among the 10,069 patients included in the study, (mean age was 60 ± 18 years, 60% male); 2511 (26.3%) of these had received a transfusion which may be attributed to the short duration of study.

Another study [40] conducted in a general hospital in Kuwait assessed transfusion practices in ICU. Of the 475 patients admitted, 99 (21%) received a blood transfusion while in the ICU which agreed with our study.

Regarding Charlson Comorbidity Index (CCI), it was higher among anemic compared to non-anemic with statistically significant difference between the two groups (P<0.05),

A cohort study by Warner et al. [41] on residents of Olmsted County, Minnesota, using medical records from the Rochester Epidemiology Project, the study included adults (aged≥18 years and older), who were admitted to ICU from January 1, 2010, through December 31, 2016. Hemoglobin levels were documented within the first 24 hours of hospitalization. The study included a total of 6901 patients, 55%of them were males and 45% were females. Non-anemic patients had lower Charlson Comorbidity Index which is similar to current study.

There was highly statistically significant increase in anemic group (P<0.001) as regards initial SOFA, mean SOFA, highest SOFA which is consistent with Vincent et al., [3] who conducted a study in European ICUs, it was reported that anemia was associated with higher (SOFA) scores.

Similarly, Juárez-Vela et al. [42] conducted an observational multicenter study of all patients admitted to ICUs across 5 Spanish hospitals. Significant variations were detected between the APACHE II and SOFA severity scales in patients with and without progression to anemia. In both cases, patients with progression to anemia had the highest scores on both scales, indicating severity of anemia.

There was highly statistically significant increase in anemic group (P<0.001) as regards APACHE II. Amer et al. [43] who conducted a prospective study among 100 critically ill elderly (≥ 60 years) admitted to the geriatric ICU at Ain shams university hospitals, it was found that the relation between the existence of anemia and APACHE II score was statistically insignificant which is against current study, However, the anemic group had a higher APACHE II score compared to the non-anemic group which may be attributed to small study sample.

Regarding outcomes among studied subjects in-hospital mortality (43.5%) were died, several studies demonstrated in hospital mortality among critically ill patients in ICU. A study of Ball et al [44] which confirmed that in hospital mortality was 41.5%, also Marik, Paul et al [45] found that ICU mortality increased with age; patients over 65years old were more than twice as likely to die as those under 45 years (36.8% vs. 14.8%). While Rellos et al. [46] conducted a prospective cohort study on 5,505 consecutive patients admitted to the ICU of a tertiary care hospital in Athens, Greece, 60 (1.1%) were in the oldest-old group (aged 90–98). It was reported that in-hospital mortality of for patients aged 90 years or older was 40%, compared to 8.9 % in those less than 90. There was statistically significant increase of length of stay and in hospital mortality in anemic group (P<0.05). It was observed that anemic group had longer length of stay and higher in-hospital mortality.

Numerous studies reported a strong association exists between anemia and poor patient outcomes across several chronic diseases. Culleton et al. [47] conducted a study in more than 12,000 older adults with normal renal function, anemia was correlated with elevated mortality rate (hazard ratio, 4.29) and hospitalization (hazard ratio, 2.16), after adjusting for age, sex, diabetes and chronic disease score.

Another study Rasmussen et al. [48] conducted a study on 222 COPD subjects admitted to ICU for the first time between 1994–2004, requiring invasive mechanical ventilation. Patient data (e.g., Charlson Comorbidity Index, hemoglobin, pH, and blood transfusions), and death were collected from medical records. Mortality was analyzed using Cox’s regression. Their finding showed that anemia in critically ill patients was associated with higher mortality which is consistent with the current study.

Also, a retrospective cohort study by Carson et al. [49] on 2083 patients The study population was (70.3%) females with a mean age of 57 years (SD, ± 17.7), found that anemia increased the risk of mortality which is the same with current study.

Similarly with our study, Song et al. [50], conducted a meta-analysis to explore the relationship between anemia and clinical outcomes. It revealed increased risk of mortality in anemic patients compared to a non-anemic population, with a significant increase in 30-day mortality. Also, Krishnasivam [51] conducted a retrospective cohort study of tertiary hospital admissions in Western Australia from July 2010 to June 2015. It included 80,765 inpatients of which 45,675 (56.55%) had anemia during admission. It was found that anemia was independently associated with increased inhospital mortality and LOS which is the same with current study.

We found that: 47.9% of in hospital mortality was among subjects with mild anemia, there was no statistically significant difference in between them (P >0.05). Amer et al [43] who conducted a prospective study among 100 critically ill elderly (≥ 60 years) admitted to the geriatric ICU at Ain shams university hospitals. It was found that there was no statistically significant relationship between anemia presence or severity and ICU outcomes, such as mortality or discharge. However, mortality was higher in the anemic group (50.8%) while discharge rate was greater among the non-anemic group (61.5%) which is consistent with current study.

The number of anemic who was mechanically ventilated was higher than non-anemic. 84.8%of anemic subjects were mechanically ventilated. 81.8% of anemic subjects were on vasopressors, there was no statistically significant difference between anemic and non-anemic (P>0.05).

Similarly, Akbaş [52] conducted a study in a nine-bed medical ICU in a tertiary-level hospital. The study involved one hundred sixty-nine patients who were admitted to the ICU between March 2016 to December 2017 and stayed in the ICU for more than 24 hours it was found that 85% of them were anemic, the low hemoglobin levels, a higher requirement for invasive mechanical ventilation, vasopressor support.

In current study 83.9% of anemic subjects had HAP, there was statistically significant increase between anemic than non-anemic (p<0.05) There is variability in results across several studies regarding incidence of hospital acquired infections among critically ill patients. This diversity can be attributed to several factors including variations in infection control protocols, coexisting comorbidities extend beyond just anemia, antibiotic exposure, usage of invasive devices, immune status of patients and length of stay.

Richards et al. [53] in the U.S. a 5-year surveillance study conducted in the medical ICU. It had been revealed that first three nosocomial infections were urinary tract infection, pneumonia and blood stream infection which is consistent with our study regarding urinary tract infection.

Vincent et al. [54] conducted a study in ICUs among 17 countries in Western Europe, excluding coronary care units as well as pediatric and special care infant units, it was found that the most common infections were pneumonia (46.9%), other infections of airways (17.8%), UTI (17.6%) and Blood stream infection (12%).

Linear regression analysis identified smoking, vasopressor use, blood transfusion, MPM 72hrs and CRP as key factors significantly impacting in-hospital mortality. Additionally, thrombocytopenia and mechanical ventilation were found to be highly significant in influencing in-hospital mortality, even when accounting for other variables.

## Conclusion

Anemia is highly prevalent among critically ill elderly patients admitted to ICU, significantly associated with in hospital mortality and extended length of stay, Thrombocytopenia and mechanical ventilation were found to be highly significant in influencing in-hospital mortality.
